# Ameliorating War’s Shadows: The Role of War-related Memories and Meta-humanization on Intergroup Reconciliation

**DOI:** 10.1177/01461672251338559

**Published:** 2025-05-21

**Authors:** Islam Borinca, Sofia Stathi, Theofilos Gkinopoulos

**Affiliations:** 1 University of Groningen, The Netherlands; 2 University College Dublin, Ireland; 3 University of Greenwich, London, UK; 4 Jagiellonian University, Krakow, Poland

**Keywords:** war-related memories, meta-dehumanization, meta-humanization, conciliatory attitudes

## Abstract

People in post-conflict settings often carry traumatic memories that exacerbate dehumanization and obstruct reconciliation. We investigated these processes through three studies in postwar Kosovo. Study 1 (*N* = 200), correlational, found that disturbing war-related memories negatively predicted openness to contact and peace with former adversaries through increased meta-dehumanization and outgroup dehumanization. Study 2 (*N* = 201), experimental, manipulated meta-humanization and revealed an interaction between disturbing war-related memories and meta-humanization (vs. meta-dehumanization and control) on openness to contact and peace, mediated by outgroup dehumanization. Study 3 (*N* = 201) replicated Study 2 and extended the interaction to competitive victimhood, showing that meta-humanization reduced competitive victimhood only for individuals with low levels of disturbing war memories. Additionally, Study 3 demonstrated that outgroup dehumanization mediated the effect of meta-humanization on all outcomes, with stronger effects for those low (vs. high) in disturbing war memories. These findings held after controlling for prior intergroup contact.

In the aftermath of wars, individuals are haunted by disturbing memories of events they experienced, witnessed, or learned about through their community. These memories manifest as flashbacks, nightmares, and avoidance of conflict-related triggers (Dashorst et al., 2020; Rogowska & Pavlova, 2023). High levels of disturbing war-related memories hinder reconciliation by reducing willingness to engage with former opponents (Figueiredo et al., 2017; Muldoon, 2024) and fostering mistrust between communities (Deblinger et al., 2011; Diamond et al., 2022; Muldoon et al., 2025). This fuels meta-dehumanization—believing the outgroup sees one’s group as less than human (Borinca, Van Assche et al., 2024; Kteily et al., 2016)—leading to further dehumanization of the outgroup (Haslam, 2015; Kteily & Landry, 2022), reduced peaceful contact, and heightened negative feelings toward outgroups. Yet, meta-humanization—the mutual recognition of shared humanity—can help counter these destructive patterns (Borinca et al., 2021; Pavetich & Stathi, 2021).

Although a body of literature has started to investigate mechanisms related to meta-(de)humanization, two critical gaps remain unexplored. First, it remains unclear whether meta-dehumanization and outgroup dehumanization follow a meaningful psychological sequence in relation to disturbing war-related memories and conciliatory attitudes (operationalized here as openness to intergroup contact, feelings of peace, and reduced competitive victimhood). We propose that individuals who report more disturbing war-related memories may also perceive that the outgroup sees their group as less than human (meta-dehumanization), which is in turn associated with more dehumanizing views of the outgroup. This sequence reflects a psychologically plausible pattern in how distress may be linked to perceptions of others and intergroup outcomes. Second, while this proposed sequence may help explain individual variation in reconciliation-related attitudes, it is also important to understand whether and for whom reconciliation-promoting strategies, such as meta-humanization, are effective. In particular, the extent to which individuals benefit from meta-humanization may depend on the intensity of their war-related memories. Addressing both questions contributes to a more nuanced understanding of how individual-level psychological factors relate to responses to (de)humanization and support for reconciliation in post-conflict contexts.

To address these gaps, we conducted three studies in post-conflict Kosovo, focusing on Kosovo Albanians who endured ethnic cleansing and atrocities by Serbian forces during the war (1998–1999; see review below). Study 1 tested a sequential mediation model examining associations between disturbing war-related memories and conciliatory attitudes through two consecutive mediators: meta-dehumanization and outgroup dehumanization. We tested this sequential mediation model because previous research suggests that perceived dehumanization relates to both past experiences of violence and intergroup attitudes (Borinca, Van Assche, et al., 2024; Kteily et al., 2016). We investigated whether the relationship between disturbing war-related memories and conciliatory attitudes is mediated through higher levels of meta-dehumanization, and in turn, higher outgroup dehumanization.

Studies 2 and 3 tested the moderating role of war-related memories on the effect of meta-humanization (compared to meta-dehumanization and control conditions) on conciliatory attitudes. We examined whether these effects would be stronger for individuals reporting lower (vs. high) levels of disturbing war-related memories, based on evidence that intergroup intervention effectiveness depends on psychological readiness to engage with the outgroup. Previous research suggests that individuals experiencing high psychological distress from past violence often display greater resistance to positive intergroup interventions (Schmid & Muldoon, 2015; Voci et al., 2017), potentially implying that such interventions may be more effective for those with lower distress. While meta-humanization might effectively promote reconciliation among the latter group, those with more disturbing war-related memories may be more resistant to the impact of meta-humanization. Finally, Studies 2 and 3 investigated whether reduced outgroup dehumanization mediated this interaction effect on conciliatory attitudes.

## Meta-humanization and Meta-dehumanization in Postwar Contexts

Research has identified several key mechanisms for improving intergroup relations, such as trust, empathy, and intergroup contact. Yet, each faces distinct challenges in postwar settings. This is because post-conflict societies are characterized by mistrust, historical grievances, and psychological barriers to reconciliation. Trust, which involves confidence in the outgroup’s intentions (Lewicki et al., 1998), remains fragile due to past violence. While empathy can foster reconciliation (Batson et al., 2002), it is difficult to elicit when groups are entrenched in victimhood narratives (Halperin, 2011). Similarly, although intergroup contact is a well-established pathway to reconciliation (Pettigrew & Tropp, 2006), physical and psychological divisions often limit its implementation (White et al., 2021). Meta-humanization offers a unique advantage by directly addressing perceptions of how the outgroup views the in-group, making it particularly relevant in contexts where cycles of dehumanization persist. Unlike trust, which requires assessing outgroup intentions, or empathy, which depends on emotional engagement, meta-humanization—the perception that one’s group is seen as fully human by the outgroup—facilitates a cognitive shift that challenges entrenched perceptions of hostility. In societies where direct contact is limited and historical grievances perpetuate hostility, changing perceptions of how one’s group is viewed by the outgroup may provide a more viable pathway to reconciliation (Borinca, Van Assche, et al., 2024; Kteily et al., 2016).

Research in post-conflict societies suggests that individuals often expect blatant dehumanization from former adversaries due to previous intergroup conflict and war (Borinca et al., 2021). This expectation, known as meta-dehumanization, refers to the belief that one’s in-group is seen as less than human by an outgroup. Regardless of its accuracy, this perception intensifies intergroup conflict by encouraging reciprocal dehumanization—where individuals dehumanize the outgroup in response (Kteily et al., 2016; Landry et al., 2022). In contrast, meta-humanization can promote reciprocal humanization, reducing intergroup bias and fostering positive relations (Borinca, Tropp, et al., 2021; Kteily et al., 2016; Moore-Berg & Hameiri, 2024; Pavetich & Stathi, 2021; Prati et al., 2023). Supporting this, both correlational and experimental studies in the Kosovo Albanian–Serbian context have demonstrated that meta-humanization decreases dehumanization, leading to greater openness to future contact and increased feelings of peace with outgroup members (Borinca, Van Assche, et al., 2024).

One critical outcome of reduced dehumanization is increased openness to intergroup contact, which is central to reconciliation (Pettigrew & Tropp, 2006). In post-conflict societies, where direct contact is often limited by physical separation and deep-seated mistrust (Çelebi et al., 2017), individuals may rely on meta-humanization to gauge whether future contact is possible and desirable (Borinca, Tropp, et al., 2021). Meta-humanization may also mitigate competitive victimhood, another key barrier to reconciliation (Noor et al., 2008). While individuals who perceive their group as suffering more than the outgroup often resist reconciliation (Schori-Eyal et al., 2017), recognizing that the outgroup sees one’s group as fully human may reduce this zero-sum perception of suffering and foster a sense of shared humanity (Pavetich & Stathi, 2021). Supporting this, research shows that normative apologies from outgroup members reduce competitive victimhood (Borinca, Koc, et al., 2025), suggesting that outgroup humanization can diminish such perceptions.

However, prior research has largely overlooked the role of war-related memories in shaping these intergroup processes. While social and political psychology have examined the influence of collective memories on reconciliation (e.g., Figueiredo et al., 2017; Pettai, 2016), interdisciplinary studies emphasize how memories shape historical knowledge, perceptions, and identity formation (Cubitt, 2018). Trauma psychology research reveals that individual war-related memories can directly influence intergroup attitudes—distressing personal memories heighten negative emotional responses, reinforce threat perceptions, and exacerbate social distance (Brewin et al., 2010; Schönfeld & Ehlers, 2017). In post-conflict societies, these memories may sustain fear, distrust, and negative outgroup expectations, deepening intergroup divisions (Hirschberger, 2018).

Taken together, the relationship between disturbing war-related memories and fundamental intergroup processes—such as meta-(de)humanization, dehumanization, and conciliatory attitudes—remains underexplored. Our research addresses this gap by examining the sequential path from war-related memories to conciliatory attitudes through meta-(de)humanization and dehumanization. Additionally, we investigate whether meta-humanization can improve conciliatory attitudes across all individuals and whether such effects are mediated via reduced dehumanization and moderated via varying levels of disturbing war-related memories.

## The Moderating Role of Disturbing War-related Memories on Conciliatory Attitudes

The level of disturbing war-related memories varies widely among individuals. While some people endure recurrent, distressing memories and intrusive thoughts with significant psychological consequences, others experience less severe recollections with fewer associated difficulties (Brewin et al., 2000; Campbell et al., 2007; Pedersen et al., 2011). These variations can influence their intergroup perceptions and behaviors toward outgroups (Mukherjee et al., 2017; Páez & Liu, 2015; Schori-Eyal et al., 2017). Drawing from trauma and autobiographical memory research (Brewin et al., 2010; Rubin et al., 2008; Schönfeld & Ehlers, 2017), we assess disturbing war-related memories across three dimensions: intensity, emotional impact, and avoidance behaviors. Intensity reflects the persistence and vividness of war-related memories. Emotional impact captures the distress associated with memory recall, a core aspect of post-traumatic distress. Avoidance behaviors—efforts to suppress or disengage from traumatic memories—influence social engagement and intergroup attitudes. Understanding how individuals with varying levels of these memories respond to meta-humanization (vs. meta-dehumanization and control conditions) is crucial for improving intergroup relations (Borinca, Sainz, et al., 2024). Moreover, examining whether meta-humanization can foster positive intergroup relations for people with both low and high levels of war-related memories may reveal important pathways to reducing conflict and hostility.

For individuals with *low levels* of disturbing war-related memories, we propose that meta-humanization will enhance conciliatory attitudes, facilitating reconciliation (Borinca, Tropp, et al., 2021; Halperin & Bar-Tal, 2011). When individuals understand that others view their group as equal human—with shared qualities, emotions, and needs—they are more likely to reciprocate by perceiving the outgroup as equally human, fostering openness to intergroup contact and peacebuilding efforts. Since they face fewer psychological barriers (i.e., less emotional distress and avoidance), meta-humanization may also effectively reduce feelings of competitive victimhood (Borinca, Koc, et al., 2025; Schmid & Muldoon, 2015; Voci et al., 2017). Critically, for those with *high levels* of disturbing war-related memories, we expect that meta-humanization will still have a positive effect, although to a lesser extent, due to the greater psychological barriers that must be overcome (Schmid & Muldoon, 2015; Voci et al., 2017). This aligns with previous research demonstrating that: (a) meta-humanization can enhance intergroup attitudes even among individuals who experience high intergroup threat, indicating that even the most vulnerable or biased individuals benefit from this strategy (Pavetich & Stathi, 2021); and (b) meta-humanization may function as a form of perceived intergroup acknowledgment, which potentially promotes psychological resilience in post-conflict settings (Muldoon & Downes, 2017; Muldoon et al., 2019, 2023). By fostering the belief that outgroup members see them as equal humans, meta-humanization may counteract the negative psychological impact of war-related memories and facilitate reconciliation.

Based on this reasoning, we hypothesized that disturbing war-related memories would moderate the effect of meta-humanization (vs. other conditions) on conciliatory attitudes. Specifically, we expected meta-humanization would reduce dehumanization, enhance openness to contact, increase feelings of peace, and mitigate competitive victimhood among individuals with both high and low levels of disturbing memories. We predicted that the effects of meta-humanization would be stronger for those with low levels of disturbing war-related memories due to decreased psychological barriers. Finally, we predicted that reduced outgroup dehumanization would mediate the interaction between war memories and meta-humanization on these outcomes.

## Kosovo’s Post-conflict Context

We conducted three studies in the postwar context of Kosovo, focusing on Kosovo Albanians who have experienced directly or indirectly (via family members or relatives) war with Serbia. Between 1998 and 1999, Serbia carried out an ethnic cleansing operation against Kosovo Albanians, resulting in the deaths of approximately 10,000 Kosovo Albanians, the rape of 20,000 people (primarily women), and the displacement of hundreds of thousands (Amnesty International, 2021; Human Rights Watch, 2001; Judah, 2008; OSCE, 2003; United Nations, 2009; U.S. Department of State, 2001). Following NATO’s intervention to end the war, Kosovo declared independence in 2008. Serbia refuses to recognize Kosovo’s independence and has neither acknowledged nor apologized for the war crimes committed during the conflict (Ker-Lindsay, 2009; Richmond et al., 2024).

Recent polls indicate that 83% of Kosovars continue to perceive Serbia as a threat, largely due to ongoing political tensions and unresolved conflict-related issues (International Republican Institute [IRI], 2023). The situation has been further complicated by recent incidents, including Kosovo’s government responses to perceived security threats from Serbian paramilitary activities. These tensions are intensified by negative perceptions and biased narratives from both sides, fostering deep mistrust and hostility (Reporting Diversity Network 2.0, 2024). This enduring conflict makes Kosovo a topical context for studying the role of war-related memories in intergroup relations.

## The Present Research

Across three studies—one cross-sectional and two experimental—we observed the relationship between disturbing war-related memories and intergroup relations in post-conflict Kosovo. Study 1 examined whether meta-dehumanization and, in turn, outgroup dehumanization sequentially mediate the association between disturbing war-related memories and conciliatory attitudes (specifically, contact orientations and feelings of peace; H1)

Building on the foundation of Study 1, Studies 2 and 3 experimentally manipulated meta-humanization to test its causal effects and examine whether these effects are moderated by individuals’ levels of disturbing war-related memories. Study 2 compared meta-humanization to both meta-dehumanization and a control condition, while Study 3 compared only the meta-humanization and meta-dehumanization conditions, omitting the control condition used in Study 2. In Study 3, we also included competitive victimhood as an outcome. In both studies, we expected a main effect of meta-humanization (vs. other conditions) on conciliatory attitudes (H2a). Additionally, we hypothesized that this effect would be moderated by levels of disturbing war-related memories, with stronger effects among individuals reporting low (vs. high) levels of such memories (H2b).

Finally, building on research showing meta-humanization’s link to reconciliation through reduced outgroup dehumanization (Borinca, Van Assche, et al., 2024; Kteily et al., 2016), we tested whether outgroup dehumanization mediates the interaction between disturbing war-related memories and meta-humanization on conciliatory attitudes (H3a). Study 3 additionally examined this mediated moderation for competitive victimhood (H3b).^1^

## Study 1

In Study 1, we examined whether meta-dehumanization and outgroup dehumanization sequentially mediate the association between disturbing war-related memories and conciliatory attitudes (specifically, contact orientations and feelings of peace).

### Methods

#### Participants and Procedure

A total of 200 Kosovo Albanians (112 women; *M*_age_ = 46.73, *SD*_age_ = 13.18) voluntarily completed a paper-and-pencil survey in Prizren, Kosovo. All participants had direct or indirect exposure to the Kosovo War (1998–1999). Due to the historical specificity of this population, further increasing the sample size was not feasible. A Monte Carlo power analysis for indirect effects (Schoemann et al., 2017) indicated sufficient power to detect key mediation effects, with estimates ranging from 0.06 to 0.79. The highest power (0.79) was observed for *a1db2* predicting feelings of peace. While larger samples are often preferable, this study offers valuable insights into reconciliation processes in post-conflict populations, where recruitment is challenging.

Participants were informed that the research aimed at understanding the traumatic experiences concerning the Kosovo War and other group members, such as Serbs. They were made aware of the sensitive nature of the topic and their right to withdraw at any time if distressed. Support helplines were provided. After giving consent, participants provided sociodemographic information, including gender, age, and nationality, and indicated whether they or their family members had experienced the Kosovo War. They could elaborate on their experiences through open-ended responses if they wished. Participants then completed measures of the study variables and were debriefed, thanked, and reminded of available helplines.

### Measures

Unless otherwise indicated, all responses were given on a seven-point Likert Scale ranging from 1 (*not at all* ) to 7 (*absolutely*).

#### Disturbing War-related Memories

We measured disturbing war-related memories using a seven-item scale. Five items were adapted from Voci et al. (2017) to assess intrusive memories of war-related events (e.g., “Repeated, disturbing memories, thoughts, or images of the Kosovo War” and “Repeated, disturbing dreams of the Kosovo War”). However, trauma research emphasizes that avoidance behaviors are also a key component of how individuals process and regulate distressing war-related memories (American Psychiatric Association [APA], 2013; Ehlers & Clark, 2000; Foa et al., 1989). Intrusive thoughts alone do not fully capture how individuals process war-related memories. Avoidance is not merely a reaction to distress but an active coping strategy that shapes psychological adjustment and intergroup perceptions (Brewin et al., 2010). By preventing full cognitive processing, avoidance can sustain distress, reinforce negative outgroup attitudes, and reduce openness to reconciliation.

Thus, to ensure a comprehensive assessment of the variable, we added two avoidance-related items: “Avoiding thinking or talking about the Kosovo War or feelings about the war” and “Avoiding situations or activities that remind one of the Kosovo War.” These items capture the extent to which individuals regulate their engagement with distressing memories, distinguishing those who suppress war-related recollections from those who confront them. The two additional items demonstrated strong internal consistency (*r* = .80; *M* = 4.18, *SD* = 2.00), and their inclusion improved overall scale reliability (α = .88, *M* = 4.35, *SD* = 1.59).^2^ Higher scores indicate greater self-reported disturbing war-related memories, suggesting that the expanded scale enhances measurement validity without altering core effects.

#### Meta-Dehumanization

We measured perceived meta-dehumanization with a five-item scale adapted from Kteily et al. (2016). Participants were asked to rate the extent to which they think Serbs dehumanize Kosovan Albanians using items such as “Serbs perceive Albanians to be subhuman” and “Serbs think of Albanians as being animal-like.” We averaged the responses to these items to compute a score for perceived meta-dehumanization (α = .92; *M* = 5.61, *SD* = 1.43). Higher scores indicated greater perceived meta-dehumanization.

#### Outgroup Dehumanization

We assessed the extent to which participants attributed traits that signal a perceived lack of human characteristics to the outgroup using a 9-item scale adapted from Kteily et al. (2016), Borinca, Van Assche, et al. (2024). This scale included traits such as *backward, primitive*, and *savage*, which are often associated with dehumanization in intergroup contexts (Bastian et al., 2013). Higher scores indicated greater dehumanization of outgroups (α = .75; *M* = 5.38, SD = 1.11).

#### Contact Orientations

We measured contact orientations with a 10-item scale adapted from Migacheva and Tropp (2013) and Pavetich and Stathi (2021). Participants were asked about their willingness to interact with Serbs using items such as “In general, how much would you like to become friends with someone who is Serbian?” and “In general, how comfortable would you be to interact with a Serb?” Higher scores indicated greater openness toward contact orientations with the outgroup (α = .87; *M* = 2.38, *SD* = 1.21).

#### Feeling at Peace With the Outgroup

We used a single item to measure participants’ feeling at peace with the outgroup, adapted from Borinca, Tropp, et al. (2021): “Do you feel at peace with Serbs?” (*M* = 1.96, *SD* = 1.54).

#### Control Measures

To control for variations in past contact with the outgroup—a key variable in shaping intergroup attitudes—we included two additional measures, each consisting of a single item. The first measure focused on direct contact, asking participants how frequently they have personally interacted with Serbs (*M* = 1.85, *SD* = 1.45). The second measure examined indirect contact, inquiring how often participants’ family and friends have had contact with Serbs (*M* = 1.62, *SD* = 1.27).^3^

### Results

We first computed zero-order Pearson correlations (Table 1) to examine relationships between disturbing war-related memories, meta-dehumanization, outgroup dehumanization, contact orientations, and feelings of peace, as well as their associations with both direct and indirect past contact experiences. Among others, results indicated that disturbing war-related memories were positively associated with meta-dehumanization, *r*(198) = .35, *p* < .001, and outgroup dehumanization, *r*(198) = .25, *p* < .001, but negatively associated with contact orientations, *r*(198) = -.28, *p* < .001, and feelings of peace, *r*(200) = −.14, *p* = .040.

**Table 1.** Correlations Among Variables (Study 1).

Measures	1	2	3	4	5	6	7
1. Disturbing war-related memories	—						
2. Meta-dehumanization	.352**	—					
3. Outgroup Dehumanization	.253**	.347**	—				
4. Contact Orientations	−.287**	−.370**	−.489**	—			
5. Feeling at peace with the outgroup	−.146*	−.321**	−.447**	.720**	—		
6. Direct contact frequency	−.211**	−.318**	−.376**	.606**	.534**	—	
7. Indirect contact frequency	−.142*	−.238**	−.372**	.597**	.563**	.749**	—

* *p* < .05. **p < .01 (two-tailed).

#### Mediation Analysis

**Figure 1.**
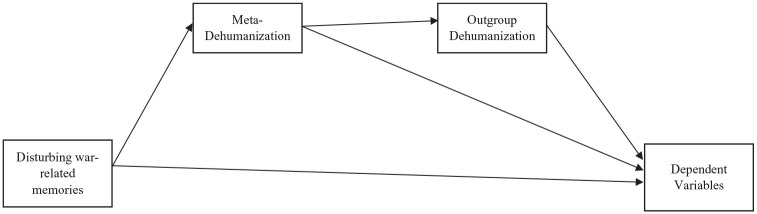
The conceptual model tested in Study 1. *Note*. The dependent variables included contact orientations and feelings of peace (Study 1).

To test our hypothesized serial mediation model (see Figure 1), we used PROCESS Model 6 (Hayes, 2018) with 5,000 bootstrapped samples. These analyses examined whether self-reported war-related memories predict contact orientations and feelings of peace with outgroups through the sequential mediators of meta-dehumanization and outgroup dehumanization. Past direct and indirect contact frequencies were included as covariates to account for prior intergroup exposure, ensuring that the observed effects primarily reflect the psychological impact of war-related memories. For contact orientations (see Table 2), there was a significant serial indirect effect from war-related memories to contact orientations via meta-dehumanization and outgroup dehumanization, *B* = −0.01, *SE* = 0.01, 95% CI [−0.03, −0.01]. Similarly, for feelings of peace with the outgroup, there was a significant serial indirect effect through the same mediators, *B* = −0.01, *SE* = 0.01, 95% CI [−0.03, −0.01].^4^

**Table 2.** Regression and Indirect Effects for the Sequential Mediation Model with Meta-dehumanization and Outgroup Dehumanization as mediators, controlling for Direct and Indirect Contacts (Study 1).

Regression Model	*B* (Coeff)	*SE*	*p*	95% CI [LL, UL]
War-related memories → Meta-dehumanization	0.27	0.06	<.001	[0.15, 0.39]
War-related memories → Outgroup dehumanization	0.08	0.05	.074	[−0.01, 0.18]
Meta-dehumanization → Outgroup dehumanization	0.17	0.05	.002	[0.06, 0.27]
Meta-dehumanization → Contact orientations	−0.09	0.05	.057	[−0.19, 0.01]
Outgroup dehumanization → Contact orientations	−0.25	0.06	<.001	[−0.37, −0.12]
Meta-dehumanization → Peace with outgroup	−0.13	0.07	.050	[−0.27, −0.00]
Outgroup dehumanization → Peace with outgroup	−0.32	0.09	<.001	[−0.49, −0.14]
Direct contact → Contact orientations	0.21	0.07	.002	[0.08, 0.34]
Indirect contact → Contact orientations	0.27	0.07	<.001	[0.12, 0.41]
Direct contact → Peace with outgroup	0.19	0.09	.042	[0.01, 0.37]
Indirect contact → Peace with outgroup	0.39	0.10	<.001	[0.19, 0.59]
Sequential indirect effects		Effect	Boot *SE*	95% CI [LL, UL]
War-related memories → Meta-dehumanization → Outgroup dehumanization → Contact orientations		−0.01	0.01	[−0.03, −0.01]
War-related memories → Meta-dehumanization → Outgroup dehumanization → Peace with outgroup		−0.01	0.01	[−0.03, −0.01]

We tested multiple alternative mediation models to explore different configurations of variables. These models consistently yielded weaker or nonsignificant indirect effects, reinforcing the rigor of our theoretical framework (see Supplemental Material).

### Discussion

In Study 1, we examined whether meta-dehumanization and outgroup dehumanization sequentially mediate the association between disturbing war-related memories and conciliatory attitudes (specifically, openness to intergroup contact and feelings of peace). Results showed that disturbing war-related memories were positively associated with meta-dehumanization, which in turn predicted higher outgroup dehumanization. This sequence was associated with reduced openness to contact and feelings of peace, even after controlling for both direct and indirect past contacts with the outgroup.

Building on these findings, Studies 2 and 3 experimentally tested whether meta-humanization (i.e., the belief that the outgroup views the in-group as human) could improve conciliatory attitudes; and whether this effect occurs for people with both high and low levels of disturbing war-related memories.

## Study 2

Study 2 experimentally tested the effects of meta-humanization by comparing it with meta-dehumanization and control conditions (see Pirlott & MacKinnon, 2016; Vázquez et al., 2021, for a similar procedure). After completing the disturbing war-related memories measure, participants were randomly assigned to one of the three conditions: meta-humanization, meta-dehumanization, or a baseline control. They then responded to measures of outgroup dehumanization, contact orientation, and feelings of peace toward the outgroup. This design allowed us to test whether the effects of meta-humanization on conciliatory attitudes (via reduced outgroup dehumanization) varied based on participants’ levels of disturbing war-related memories.

### Methods

#### Participants and Procedure

As in Study 1, we recruited 201 Kosovan Albanian participants (87 women; *M*age = 49.39, *SD*age = 12.31) as part of a field study conducted in the region of Peja in Kosovo. All participants reported having experienced the Kosovo War either directly or via family members. A sensitivity analysis conducted with G*Power (ver. 3.1.9.2) for ANCOVA (fixed effects model) revealed that our final sample provided adequate power to detect a medium-sized main effect or interaction effect (*f* = 0.29), assuming an α value of .05 and a power estimate of .80 (Faul et al., 2009).

#### Procedure

Participants were informed that the research examined traumatic experiences from the Kosovo War and intergroup relations. They were provided with helpline information before beginning participation, in case any part of the survey caused distress. After providing informed consent, participants completed a paper-and-pencil questionnaire in two sections: first, demographic information and disturbing war-related memories measures, then the experimental manipulation, followed by dependent variables. Upon completion, participants received a detailed written debrief explaining the study’s purpose and use of deception. An experimenter was present to address any concerns, and participants could respond either verbally or in writing. They were explicitly asked to reaffirm their consent, with a reminder about available helplines in case of emotional distress. No participants withdrew from the study after debriefing.

### Measures and Experimental Manipulation

We assessed participants’ self-reported disturbing war-related memories (α = .85; *M* = 4.41, *SD* = 1.30) using the same scale as in Study 1 (Voci et al., 2017). Each participant was then randomly assigned to one of the three experimental conditions—meta-dehumanization, meta-humanization, or control—based on the questionnaire they received (e.g., Borinca, Tropp, et al., 2021; Kteily et al., 2016). To reduce the possibility of social desirability bias, participants were informed that the content presented in the experimental conditions was derived from international research before receiving any information.

Participants in the meta-dehumanization (*n* = 67) and meta-humanization (*n* = 67) conditions read a brief excerpt from a bogus scientific article stating that Serbs rated themselves as highly developed and civilized (96 out of 100 points). Depending on the condition, participants then learned that Serbs rated Kosovo Albanians as either equally evolved and civilized (96 out of 100; meta-humanization condition) or less evolved and civilized (67 out of 100; meta-dehumanization condition). Participants in the baseline control condition (*n* = 67) did not receive any information.

#### Dependent Variables

Finally, participants completed the same measures as in Study 1: outgroup dehumanization (α = .89; *M* = 4.47, *SD* = 1.40), contact orientations (α = .87; *M* = 3.86, *SD* = 1.31), and feelings of peace (*M* = 3.46, *SD* = 1.87). As a manipulation check, we used meta-dehumanization (α = .92; *M* = 4.50, *SD* = 1.74) to assess the effectiveness of our experimental manipulation.

##### Control Measures

As in Study 1, we assessed both direct (*M* = 2.56, *SD* = 1.10) and indirect past contacts with Serbs (*M* = 1.93, *SD* = 1.22).

### Results

To test our hypotheses, we computed two orthogonal contrasts, a method offering more precise analysis for variables with more than two categories (Borinca, Guerra, et al., 2024; Brauer & McClelland, 2005; Furr & Rosenthal, 2003). The first contrast (C1) compared the meta-humanization condition (+2) against the meta-dehumanization and control conditions (−1 each). The second contrast (C2) compared meta-dehumanization (+1) and control (−1) conditions, with meta-humanization coded as 0. A linear effect is indicated when C1 is significant, but not C2. We entered these contrasts, standardized disturbing war-related memories, and their interactions (except between contrasts) as independent variables in a full factorial ANCOVA, controlling for both direct and indirect past contacts. This contrast coding approach was based on prior research in similar post-conflict contexts (e.g., Borinca, Tropp, et al., 2021; Borinca, Van Assche, et al., 2024), which found no significant differences between meta-dehumanization and control conditions across various intergroup outcomes, including outgroup perceptions, intergroup attitudes, and behavioral intentions. These findings suggest that in post-conflict settings like Kosovo, where historical grievances and ongoing tensions persist, baseline intergroup perceptions often align with expectations of negative treatment by the outgroup, making the control condition comparable to the meta-dehumanization condition. Table 3 presents the estimated means and standard errors for all dependent variables.

**Table 3.** The Interactive Effect of War-related Memories and Meta-humanization (vs. other conditions) on Our Investigated Outcomes in Studies 2 and 3.

Study 2 (*N* = 201)	Disturbing War-related Memories					
	Low (−1 *SD*)			High (+1 *SD*)		
	Experimental Manipulation			Experimental Manipulation		
	Meta-Humanization	Meta-Dehumanization	Control	Meta-Humanization	Meta-Dehumanization	Control
Dehumanization	3.12 (0.25)^a^	5.53 (0.27)^b^	5.58 (0.24)^b^	4.55 (0.23)^a^	5.67 (0.25)^b^	5.57 (0.21)^b^
Contact orientations	4.56 (0.23)^a^	2.20 (0.25)^b^	2.90 (0.26)^b^	3.76 (0.21)^a^	2.53 (0.23)^b^	2.95 (0.23)^b^
Feeling at peace	4.26 (0.38)^a^	1.53 (0.41)^b^	2.73 (0.42)^b^	3.28 (0.35)^a^	1.63 (0.39)^b^	2.69 (0.36)^b^
Experiment 3 (*N* = 201)	Disturbing War-related Memories					
	Low (−1 *SD*)			High (+1 *SD*)		
	Experimental Manipulation			Experimental Manipulation		
	Meta-humanization	Meta-dehumanization		Meta-humanization	Meta-dehumanization	
Dehumanization	2.57 (0.16)^a^	4.42 (0.25)^b^		5.06 (0.25)^a^	5.60 (0.19)^b^	
Contact Orientations	5.14 (0.17)^a^	3.81 (0.27)^b^		3.49 (0.27)^a^	2.96 (0.20)^b^	
Feeling at peace	5.39 (0.27)^a^	3.50 (0.43)^b^		3.24 (0.43)^a^	2.50 (0.26)^b^	
Competitive Victimhood	3.08 (0.21)^a^	5.54 (0.35)^b^		6.58 (0.35)^a^	6.41 (0.26)^a^	

*Note*. Means and standard errors (in parentheses) for experimental manipulation at conditional levels (low vs. high) of disturbing war-related memories. Means with different letters differ at least at *p* < .05.

#### Manipulation Check

The manipulation check confirmed that our experimental manipulation effectively influenced perceptions of meta-(de)humanization. Participants in the meta-humanization condition reported lower meta-dehumanization (*M* = 3.40, *SD* = 1.69) than those in the meta-dehumanization (*M* = 4.97, *SD* = 1.48) and control conditions (*M* = 5.14, *SD* = 1.49), as indicated by a significant contrast, *F*(1, 195) = 40.03, *p* < .001, *η²* = .17.

Additionally, the main effect of disturbing war-related memories was significant, *F*(1, 195) = 4.05, *p* = .045, *η²* = .02, showing that meta-dehumanization increased as disturbing war-related memories increased (*B* = 0.26). No other effects reached significance, all *F_s* < 1.40, all *p_s* > .237.

#### Outgroup Dehumanization

The main effect of disturbing war-related memories was significant, *F*(1, 195) = 10.63, *p* < .001, *η²* = .05: Outgroup dehumanization increased as disturbing war-related memories increased (*B* = 0.24). The predicted C1 was also significant, *F*(1, 195) = 168.45, *p* < .001, *η²* = .46, showing that participants in the meta-humanization condition reported lower outgroup dehumanization (*M* = 3.13, *SD* = 1.21) than those in meta-dehumanization (*M* = 5.16, *SD* = 0.96) and control conditions (*M* = 5.14, *SD* = 0.87). However, C2 was not significant, *F*(1, 195) = 0.001, *p* = .991, *η²* = .001. The predicted C1 × disturbing war-related memories interaction was significant, *F*(1, 195) = 20.57, *p* < .001, *η²* = .09, while the C2 × disturbing war-related memories interaction was not significant, *F*(1, 195) = 1.73, *p* = .190, *η²* = .009.

Simple effects analysis of the C1 × disturbing war-related memories interaction revealed that meta-humanization (vs. meta-dehumanization and control conditions) reduced outgroup dehumanization for participants with both low (−1 *SD*), *F*(1, 195) = 189.02, *p* < .001, *η²* = .49 and high (+1 *SD*), *F*(1, 195) = 29.90, *p* < .001, *η²* = .13 levels of disturbing war-related memories. Notably, in line with our hypotheses, the magnitude of this effect was greater for individuals with low levels of disturbing war-related memories than for those with high levels.

#### Contact Orientations

The main effect of disturbing war-related memories was not significant, *F*(1, 195) = 1.33, *p* = .716, *η²* = .001. The predicted C1 was significant, *F*(1, 195) = 115.29, *p* < .001, *η²* = .37, showing that participants in the meta-humanization condition reported greater willingness for intergroup contact (*M* = 5.01, *SD* = 1.08) than those in meta-dehumanization (*M* = 3.08, *SD* = 0.79) and control conditions (*M* = 3.49, *SD* = 1.16). C2 was also significant, *F*(1, 195) = 8.85, *p* = .003, *η²* = .04, indicating lower contact willingness in the meta-dehumanization condition compared to control. The predicted C1 × disturbing war-related memories interaction was significant, *F*(1, 195) = 11.84, *p* < .001, *η²* = .05, while the C2 × disturbing war-related memories interaction was not significant, *F*(1, 195) = 0.47, *p* = .492, *η²* = .002.

Simple effects analysis of the C1 × disturbing war-related memories interaction revealed that meta-humanization (vs. meta-dehumanization and control conditions) increased contact willingness for participants with both low (−1 *SD*), *F*(1, 195) = 111.36, *p* < .001, *η²* = .36 and high (+1 *SD*), *F*(1, 195) = 24.66, *p* < .001, *η²* = .13 levels of disturbing war-related memories. Notably, the magnitude of this effect was greater for individuals with low levels of disturbing war-related memories than for those with high levels.

#### Feeling at Peace

The main effect of disturbing war-related memories was not significant, *F*(1, 195) = 0.98, *p* = .149, *η²* = .01. The predicted C1 was significant, *F*(1, 195) = 65.91, *p* < .001, *η²* = .25, showing that participants in the meta-humanization condition reported greater feelings of peace (*M* = 4.87, *SD* = 1.69) than those in meta-dehumanization (*M* = 2.48, *SD* = 1.23) and control conditions (*M* = 3.04, *SD* = 1.74). C2 was also significant, *F*(1, 195) = 5.43, *p* = .021, *η²* = .02, indicating lower feelings of peace in the meta-dehumanization condition compared to control. The predicted C1 × disturbing war-related memories interaction was significant, *F*(1, 195) = 5.42, *p* = .020, *η²* = .02, while the C2 × disturbing war-related memories interaction was not significant, *F*(1, 195) = 0.01, *p* = .910, *η²* = .001.

Simple effects analysis of the C1 × disturbing war-related memories interaction revealed that meta-humanization (vs. meta-dehumanization and control conditions) increased feelings of peace for participants with both low (−1 *SD*), *F*(1, 195) = 62.51, *p* < .001, *η²* = .24 and high (+1 *SD*), *F*(1, 195) = 15.20, *p* < .001, *η²* = .07 levels of disturbing war-related memories. Notably, the magnitude of this effect was greater for individuals with low levels of disturbing war-related memories than for those with high levels.

#### Mediation Analysis

**Figure 2.**
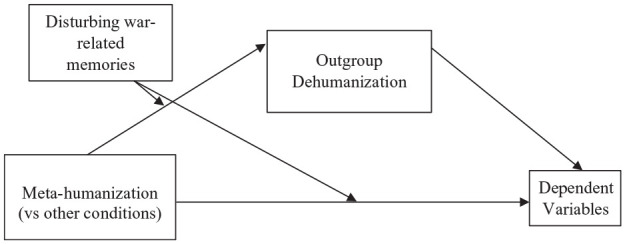
The conceptual model tested in Studies 2 and 3. *Note*. In Study 2, meta-humanization was compared with both meta-dehumanization and control (baseline) conditions, whereas in Study 3, meta-humanization was compared with meta-dehumanization only. The dependent variables included contact orientations and feelings of peace (Studies 2 and 3) and competitive victimhood (Study 3).

To test H3a, we conducted moderated mediation analyses using PROCESS for SPSS (Model 8; Hayes, 2018; 5,000 bootstrapped samples) with contact orientations and feelings of peace as outcomes. We entered either C1 (meta-humanization vs. meta-dehumanization and control) or C2 (meta-dehumanization vs. control) as the independent variable, disturbing war-related memories as a moderator, and outgroup dehumanization as mediator (see Borinca, Van Assche, et al., 2024; Valsecchi et al., 2024), controlling for both direct and indirect past contacts with Serbs (see Figure 2). See Table 4 for all direct and indirect effects.

**Table 4.** Regression and Conditional Indirect Effects of Disturbing War-related Memories and Meta-humanization (vs. Meta-dehumanization and Control) via Dehumanization on Contact Orientations and Feelings of Peace (Study 2).

Regression Model	*B*	*SE*	*p*	LLCI	ULCI
Outcome variable: Dehumanization					
Constant	5.11	0.16	<.001	4.79	5.43
C1	−0.4	0.03	<.001	−0.50	−0.36
War-related memories	0.18	0.06	.008	0.04	0.31
Interaction	0.17	0.03	<.001	0.11	0.24
Direct contact	−0.02	0.07	.677	−0.16	0.10
Indirect contact	−0.18	0.06	.003	−0.30	−0.05
Outcome variable: Contact orientations					
Constant	5.25	0.40	<.001	4.45	6.04
C1	0.20	0.04	<.001	0.10	0.29
Dehumanization	−0.42	0.07	<.001	−0.57	−0.28
War-related memories	0.04	0.06	.486	−0.08	0.18
Interaction	−0.04	0.03	.232	−0.11	0.02
Direct contact	0.13	0.07	.057	−0.03	0.27
Indirect contact	0.05	0.06	.366	−0.06	0.18
Outcome variable: Feelings of peace					
Constant	5.07	0.67	<.001	3.75	6.39
C1	0.26	0.07	.007	0.11	0.41
Dehumanization	−0.46	0.11	.001	−0.70	−0.23
War-related memories	−0.04	0.11	.721	−0.27	0.18
Interaction	−0.05	0.05	.426	−0.16	0.06
Direct contact	0.07	0.11	.551	−0.16	0.30
Indirect contact	0.10	0.10	.346	−0.11	0.31
Conditional indirect effects of meta-humanization (vs. meta-dehumanization and control) on contact orientations via dehumanization at different levels of war-related memories					
		Effect	Boot *SE*	LLCI	ULCI
Index		−0.07	0.01	−0.11	−0.04
−1.07 (−1 *SD* below average)		0.26	0.05	0.16	0.38
0.11 (average)		0.17	0.03	0.10	0.25
1.00 (+1 *SD* above average)		0.11	0.02	0.05	0.17
Conditional indirect effects of meta-humanization (vs. meta-dehumanization and control) on peace feelings via dehumanization at different levels of war-related memories					
		Effect	Boot *SE*	LLCI	ULCI
Index		−0.08	0.02	−0.14	−0.03
−1.07 (−1 *SD* below average)		0.29	0.08	0.13	0.47
0.11 (average)		0.19	0.05	0.18	0.31
1.00 (+1 *SD* above average)		0.12	0.04	0.04	0.21

For contact orientations, the moderated mediation index (−0.05) was significant, 95% CI [−0.11, −0.04]. The indirect effect of C1 was significant at both low levels of disturbing war-related memories, *B* = 0.26 (bootstrapped *SE* = 0.05), 95% CI [0.16, 0.38], and high levels of disturbing war-related memories, *B* = 0.11 (bootstrapped *SE* = 0.01), 95% CI [0.05, 0.17]. The moderated mediation index for C2 was not significant, 95% CI [−0.26, 0.08].

For feelings of peace, the moderated mediation index (−0.08) was significant, 95% CI [−0.14, −0.03]. The indirect effect of C1 was significant at both low levels of disturbing war-related memories, *B* = 0.29 (bootstrapped *SE* = 0.08), 95% CI [0.13, 0.47], and high levels of disturbing war-related memories, *B* = 0.12 (bootstrapped *SE* = 0.04), 95% CI [0.04, 0.21]. The moderated mediation index for C2 was not significant, 95% CI [−0.30, 0.10].

To assess the robustness of our findings, we tested alternative models using PROCESS Model 8, examining different mediation structures to explore the interplay between war-related memories and meta-humanization in shaping conciliatory attitudes (see Supplemental Material). The original model, which included dehumanization as the mediator, provided the strongest explanatory power. While alternative pathways through contact orientations and feelings of peace showed some indirect effects, these effects were consistently weaker, with some failing to reach significance. These findings support our theory-driven model by reinforcing dehumanization as the key psychological mechanism linking the interaction between war-related memories and meta-humanization to conciliatory attitudes.

### Discussion

Study 2 provided novel evidence on the interaction between disturbing war-related memories and meta-humanization. Compared to other conditions, meta-humanization reduced outgroup dehumanization and increased willingness for contact and feelings of peace with the former enemy, with stronger effects for individuals reporting low levels of disturbing war-related memories. Additionally, outgroup dehumanization mediated the interaction’s effects on contact orientations and feelings of peace. Specifically, meta-humanization reduced outgroup dehumanization, which in turn predicted greater openness to contact and peace across all levels of disturbing memories, though these effects were stronger for those with low levels. These findings held while controlling for both actual direct and indirect contacts with Serbs.

Building on these results, Study 3 examined whether this interaction could also reduce competitive victimhood, a well-known barrier to reconciliation (Noor et al., 2008; Vollhardt, 2012; Vollhardt et al., 2021), while seeking to replicate our findings on conciliatory attitudes.

## Study 3

As in Study 2, we first assessed disturbing war-related memories as an individual difference variable and then experimentally manipulated meta-humanization versus meta-dehumanization. Based on prior research and findings from Study 2 indicating that baseline perceptions often align with meta-dehumanization in this context, we retained the meta-dehumanization condition and omitted the control condition in Study 3. This decision allowed us to focus on testing the relative effects of meta-humanization versus meta-dehumanization on reconciliation outcomes (Borinca, Tropp, et al., 2021; Borinca, Van Assche, et al., 2024). While maintaining the same dependent variables as in Studies 1 and 2, we added competitive victimhood as an outcome (e.g., Borinca, Koc, et al., 2025; Shnabel et al., 2013). This allowed us to examine whether meta-humanization could reduce competitive victimhood through decreased outgroup dehumanization among individuals with varying levels of disturbing war-related memories. Consistent with Study 2, we expected stronger effects for individuals with low levels of disturbing memories.

### Method

#### Participants and Procedure

As in previous studies, we recruited 201 Kosovar Albanian participants (97 women; *M*_age_ = 51.29, *SD*_age_ = 10.20) as part of a field study conducted in the regions of Gjakova and Klina in Kosovo. All participants reported having experienced the Kosovo War either directly or via family members. A sensitivity analysis conducted with G*Power (ver. 3.1.9.2) for ANCOVA (fixed effects model) revealed that our final sample provided adequate power to detect a medium-sized main effect or interaction effect (*f* = 0.29), assuming an α value of .05 and a power estimate of .80 (Faul et al., 2009).

### Measures and Experimental Manipulation

We first assessed participants’ self-reported disturbing war-related memories (α = .94; *M* = 4.45, *SD* = 1.79) and then randomly assigned them to one of the two experimental conditions—meta-dehumanization (*n* = 100) and meta-humanization (*n* = 101), following the procedure from Study 2.

#### Dependent Variables

Participants completed the same measures as in Study 2: outgroup dehumanization (α = .94; *M* = 4.44, *SD* = 1.70), contact orientations (α = .95; *M* = 3.83, *SD* = 1.38), feelings of peace (*M* = 3.60, *SD* = 1.98), and manipulation check items (α = .92; *M* = 4.38, *SD* = 1.82). Finally, we assessed competitive victimhood using a three-item scale adapted from Shnabel et al. (2013; e.g., “Kosovars have experienced more terrible atrocities than Serbs,” and “Kosovars have experienced emotional pain more than Serbs”; α = .95; *M* = 4.91, *SD* = 2.05).

##### Control Measures

As in previous studies, we assessed both direct (*M* = 1.98, *SD* = 0.95) and indirect past contacts (*M* = 1.74, *SD* = 1.07).

### Results

We entered standardized disturbing war-related memories, the experimental manipulation (meta-humanization = −1, meta-dehumanization = +1), and their interaction term as independent variables in a full factorial ANCOVA. Outgroup dehumanization, contact orientations, feelings of peace, and competitive victimhood served as dependent variables, while controlling for both direct and indirect contacts. Table 3 provides the estimated means and standard errors for all dependent variables.

#### Manipulation Check

The manipulation check indicated that the main effect of disturbing war-related memories was significant, *F*(1, 197) = 64.08, *p* < .001, *η²* = .24: meta-dehumanization increased as disturbing war-related memories increased (*B* = .89). The main effect of experimental manipulation was also significant, *F*(1, 197) = 20.37, *p* < .001, *η²* = .09, showing that participants in the meta-humanization condition reported lower meta-dehumanization (*M* = 3.40, *SD* = 1.82) than those in the meta-dehumanization condition (*M* = 5.36, *SD* = 1.18). The interaction between experimental manipulation and disturbing war-related memories was also significant, *F*(1, 197) = 21.22, *p* < .001, *η²* = .09.

Simple effects analysis revealed that for participants with low levels of disturbing war-related memories (−1 *SD*), the meta-humanization condition (*M* = 2.86, *SE* = 0.22) significantly reduced meta-dehumanization compared to the meta-dehumanization condition (*M* = 4.82, *SE* = 0.36), *F*(1, 197) = 36.50, *p* < .001, *η²* = .15. In contrast, participants with high levels of disturbing war-related memories (+1 *SD*) demonstrated no significant difference in meta-dehumanization between meta-humanization (*M* = 5.66, *SE* = 0.36) and meta-dehumanization conditions (*M* = 5.60, *SE* = 0.27), *F*(1, 197) = 0.04, *p* = .833, *η²* = .001.

#### Outgroup Dehumanization

The main effect of disturbing war-related memories was significant, *F*(1, 197) = 132.25, *p* < .001, *η²* = .40: outgroup dehumanization increased as disturbing war-related memories increased (*B* = 0.91). The predicted experimental manipulation’s main effect was also significant, *F*(1, 197) = 62.26, *p* < .001, *η²* = .24, showing that participants in the meta-humanization condition reported lower outgroup dehumanization (*M* = 3.30, *SD* = 1.58) than those in the meta-dehumanization condition (*M* = 5.56, *SD* = 0.85). The predicted interaction between experimental manipulation and disturbing war-related memories was also significant, *F*(1, 197) = 18.02, *p* < .001, *η²* = .08.

Simple effects analysis revealed that meta-humanization (vs. meta-dehumanization) reduced outgroup dehumanization for participants with both low (−1 *SD*), *F*(1, 197) = 63.84, *p* < .001, *η²* = .24 and high (+1 *SD*), *F*(1, 197) = 6.71, *p* = .010, *η²* = .03 levels of disturbing war-related memories. Notably, the magnitude of this effect was greater for individuals with low levels of disturbing war-related memories than for those with high levels.

#### Contact Orientations

The main effect of disturbing war-related memories was significant, *F*(1, 197) = 55.20, *p* < .001, *η²* = .22: contact orientations decreased as disturbing war-related memories increased (*B* = -.62). The predicted experimental manipulation’s main effect was also significant, *F*(1, 197) = 33.09, *p* < .001, *η²* = .14, showing that participants in the meta-humanization condition reported greater willingness for intergroup contact (*M* = 4.66, *SD* = 1.20) than those in the meta-dehumanization condition (*M* = 3.01, *SD* = 0.99). The predicted interaction between experimental manipulation and disturbing war-related memories was also significant, *F*(1, 197) = 5.60, *p* = .019, *η²* = .02.

Simple effects analysis revealed that meta-humanization (vs. meta-dehumanization) increased contact willingness for participants with both low (−1 *SD*), *F*(1, 197) = 28.44, *p* < .001, *η²* = .12 and high (+1 *SD*), *F*(1, 197) = 6.02, *p* = .015, *η²* = .03 levels of disturbing war-related memories. Notably, in line with our hypotheses, the magnitude of this effect was greater for individuals with low levels of disturbing war-related memories than for those with high levels.

#### Feeling at Peace

The main effect of disturbing war-related memories was significant, *F*(1, 197) = 34.29, *p* < .001, *η²* = .15: feelings of peace decreased as disturbing war-related memories increased (*B* = −0.78). The predicted experimental manipulation’s main effect was significant, *F*(1, 197) = 26.76, *p* < .001, *η²* = .15, showing that participants in the meta-humanization condition reported greater feelings of peace (*M* = 4.72, *SD* = 1.80) than those in the meta-dehumanization condition (*M* = 2.50, *SD* = 1.50). The predicted interaction between experimental manipulation and disturbing war-related memories was also significant, *F*(1, 197) = 4.96, *p* = .032, *η²* = .02.

Simple effects analysis revealed that meta-humanization (vs. meta-dehumanization) increased feelings of peace for participants with both low (−1 *SD*), *F*(1, 197) = 23.24, *p* < .001, *η²* = .10 and high (+1 *SD*), *F*(1, 197) = 4.74, *p* = .031, *η²* = .02 levels of disturbing war-related memories. Notably, the magnitude of this effect was greater for individuals with low levels of disturbing war-related memories than for those with high levels.

#### Competitive Victimhood

The main effect of disturbing war-related memories was significant, *F*(1, 197) = 102.34, *p* < .001, *η²* = .34: competitive victimhood increased as disturbing war-related memories increased (*B* = 1.09). The predicted experimental manipulation’s main effect was also significant, *F*(1, 197) = 31.61, *p* < .001, *η²* = .14, showing that participants in the meta-humanization condition reported lower competitive victimhood (*M* = 3.71, *SD* = 2.16) than those in the meta-dehumanization condition (*M* = 6.10, *SD* = 0.95). The predicted interaction between experimental manipulation and disturbing war-related memories was also significant, *F*(1, 197) = 38.00, *p* < .001, *η²* = .16.

Simple effects analysis revealed that meta-humanization (vs. meta-dehumanization) reduced competitive victimhood for participants with low levels, (−1 *SD*), *F*(1, 197) = 61.07, *p* < .001, *η²* = .23, but not for those with high levels, (+1 *SD*), *F*(1, 197) = 0.35, *p* = .553, *η²* = .002, of disturbing war-related memories.

#### Mediation Analysis

To test H3b, we conducted moderated mediation analyses using PROCESS for SPSS (Model 8; Hayes, 2018; 5,000 bootstrapped samples) with contact orientations, feelings of peace, and competitive victimhood as outcomes. We entered either the experimental manipulation (meta-humanization = −1, meta-dehumanization = +1) as the independent variable, disturbing war-related memories as a moderator, and outgroup dehumanization as a mediator (see Figure 2), controlling for both direct and indirect contacts. See Table 5 for all direct and indirect effects.

**Table 5.** Regression and Conditional Indirect Effects of Disturbing War-related Memories and Meta-humanization (vs. Meta-dehumanization) via Dehumanization on Contact Orientations, Feelings of Peace, and Competitive Victimhood (Study 3).

Regression Model	*B*	*SE*	*p*	LLCI	ULCI
Outcome variable: Dehumanization					
Constant	4.41	0.17	<.001	4.06	4.76
Experimental variable	0.59	0.07	<.001	0.44	0.74
War-related memories	0.91	0.07	<.001	0.75	1.07
Interaction	−0.33	0.07	<.001	−0.48	−0.17
Direct contact	0.18	0.07	.011	0.04	0.33
Indirect Contact	−0.09	0.06	.135	−0.21	0.02
Outcome variable: Contact orientations					
Constant	6.23	0.33	<.001	5.58	6.89
Experimental variable	−0.13	0.07	.080	−0.29	0.01
Dehumanization	−0.53	0.06	<.001	−0.66	−0.40
War-related memories	−0.13	0.09	.157	−0.32	0.05
Interaction	0.01	0.07	.815	−0.13	0.16
Direct contact	−0.07	0.07	.901	−0.14	0.12
Indirect contact	0.07	0.05	.989	−0.11	0.11
Outcome variable: Feelings of peace					
Constant	7.13	0.54	<.001	6.07	8.20
Experimental variable	−0.18	0.12	.146	−0.44	0.06
Dehumanization	−0.78	0.10	<.001	−0.99	−0.57
War-related memories	−0.06	0.15	.675	−0.36	0.23
Interaction	0.02	0.12	.844	−0.21	0.26
Direct contact	0.03	0.11	0.73	−0.18	0.25
Indirect contact	−0.07	0.09	0.44	−0.25	0.11
Outcome variable: Competitive victimhood					
Constant	3.25	0.45	<.001	2.34	4.15
Experimental variable	0.28	0.10	.010	0.06	0.50
Dehumanization	0.48	0.09	<.001	0.30	0.66
War-related memories	0.64	0.13	<.001	0.38	0.90
Interaction	−0.49	0.10	<.001	−0.69	−0.28
Direct contact	0.03	0.09	.961	−0.18	0.19
Indirect contact	−0.13	0.07	0.08	−0.29	0.01
Conditional indirect effects of meta-humanization (vs. meta-dehumanization) on contact orientations via dehumanization at different levels of war-related memories					
		Effect	Boot *SE*	LLCI	ULCI
Index		0.17	0.05	0.06	0.29
−1.36 (−1 *SD* below average)		−0.56	0.13	−0.82	−0.30
0.22 (average)		−0.27	0.06	−0.41	−0.15
0.94 (+1 *SD* above average)		−0.15	0.06	−0.29	−0.03
Conditional indirect effects of meta-humanization (vs. meta-dehumanization) on peace feelings via dehumanization at different levels of war-related memories					
		Effect	Boot *SE*	LLCI	ULCI
Index		0.26	0.08	0.10	0.44
−1.36 (−1 *SD* below average)		−0.82	0.19	−1.23	−0.46
0.22 (average)		−0.40	0.09	−0.61	−0.23
0.94 (+1 *SD* above average)		−0.22	0.09	−0.42	−0.04
Conditional indirect effects of meta-humanization (vs. meta-dehumanization) on competitive victimhood via dehumanization at different levels of war-related memories					
		Effect	Boot *SE*	LLCI	ULCI
Index		−0.16	0.05	−0.22	−0.06
−1.36 (−1 *SD* below average)		0.51	0.12	0.26	0.76
0.22 (average)		0.25	0.05	0.13	0.37
0.94 (+1 *SD* above average)		0.13	0.05	0.02	0.25

For contact orientations, the moderated mediation index (0.17) was significant, 95% CI [0.06, 0.29]. The indirect effect was significant at both low levels of disturbing war-related memories, *B* = −0.56 (bootstrapped *SE* = 0.13), 95% CI [−0.82, −0.30], and high levels, *B* = −0.15 (bootstrapped *SE* = 0.06), 95% CI [−0.29, −0.03], though stronger for those with low levels. For feelings of peace, the moderated mediation index (0.26) was significant, 95% CI [0.10, 0.44]. The indirect effect was significant at both low, *B* = −0.82 (bootstrapped *SE* = 0.19), 95% CI [−1.23, −0.44], and high levels of disturbing war-related memories, *B* = −0.22 (bootstrapped *SE* = 0.09), 95% CI [−0.42, −0.04], though stronger for those with low levels. For competitive victimhood, the moderated mediation index (−0.16) was significant, 95% CI [−0.22, −0.05]. The indirect effect was significant at both low, *B* = 0.51 (bootstrapped *SE* = 0.12), 95% CI [0.26, 0.76], and high levels of disturbing war-related memories, *B* = 0.13 (bootstrapped *SE* = 0.05), 95% CI [0.02, 0.25], though stronger for those with low levels.

To assess robustness, we tested alternative models using PROCESS Model 8 with different mediation structures (see Supplemental Material). The results confirmed that our theory-driven model—with dehumanization as the mediator—provided the best fit to the data. Alternative pathways showed weaker or nonsignificant indirect effects, reinforcing dehumanization as the key psychological mechanism linking war-related memories and meta-humanization to conciliatory attitudes.

### Discussion

Study 3 replicated and extended our previous findings on the interaction between disturbing war-related memories and meta-humanization. Compared to meta-dehumanization, meta-humanization reduced outgroup dehumanization and increased contact willingness and feelings of peace for participants with both low and high levels of disturbing war-related memories, though effects were stronger for those with low levels.

Study 3 also revealed that meta-humanization reduced competitive victimhood, but only for participants with low levels of disturbing memories. Those with high levels reported higher competitive victimhood regardless of condition, suggesting that deeply affecting war-related memories may create resistance to changing perceptions of in-group suffering relative to the outgroup, even when the outgroup is believed to humanize the in-group. Notably, outgroup dehumanization mediated meta-humanization’s effects on all outcomes for both groups, indicating that while meta-humanization did not directly reduce competitive victimhood for those with high levels of disturbing memories, it had an indirect effect through reduced dehumanization, albeit weaker than for those with low levels.

## General Discussion

Across three studies, we investigated if and how disturbing war-related memories shape intergroup reconciliation, and whether meta-humanization could promote positive intergroup outcomes across different levels of these memories. Our findings consistently demonstrated the effectiveness of meta-humanization in promoting conciliatory attitudes, even when controlling for direct and indirect contacts with former adversaries.

Study 1 revealed that higher levels of disturbing war-related memories were associated with increased meta-dehumanization, leading to greater outgroup dehumanization and reduced conciliatory attitudes. Studies 2 and 3 extended these correlational findings by experimentally testing how individuals with varying levels of war-related memories respond to meta-humanization, a process that has proven effective in reducing intergroup hostility. We measured war-related memories before the experimental manipulation, allowing us to examine how preexisting trauma levels shape responses to reconciliation interventions. Both experimental studies showed that meta-humanization improved conciliatory attitudes through reduced outgroup dehumanization for participants with both low and high levels of disturbing memories, though effects were consistently stronger for those with low levels. Study 3 further revealed that meta-humanization reduced competitive victimhood, but only for those with low levels of disturbing memories. Importantly, outgroup dehumanization emerged as the key mechanism linking the interaction between war-related memories and meta-humanization to all outcomes.

While previous research has demonstrated that disturbing war-related memories and exposure to traumatic events can profoundly impact both individuals (Dashorst et al., 2020; Rogowska & Pavlova, 2023) and group dynamics (Muldoon, 2024; Muldoon et al., 2025), our research extends these findings by revealing how such memories impair intergroup reconciliation through increased perceptions of meta-dehumanization and, in turn, outgroup dehumanization, ultimately reducing contact orientations and feelings of peace.

Our results extend the literature on meta-humanization strategies. Prior research has shown that meta-humanization, compared to meta-dehumanization or control conditions, improves intergroup relations through reduced outgroup dehumanization (Borinca, Tropp, et al., 2021; Borinca, Van Assche, et al., 2024) and benefits individuals experiencing high intergroup threat (Pavetich & Stathi, 2021). Our research advances this understanding by demonstrating that meta-humanization—both directly and through reduced outgroup dehumanization—promotes conciliatory attitudes toward former adversaries across varying levels of disturbing war-related memories. This is critical when seeking to understand how to enhance reconciliation in postwar contexts, while considering the fundamental role of individual differences in this process.

Our findings also contribute to understanding competitive victimhood (Noor et al., 2008; Vollhardt et al., 2021). While meta-humanization promotes conciliatory attitudes even among those with high war-related memories, reducing competitive victimhood appears to require both mutual human recognition and decreased outgroup dehumanization. Survey data regarding the Kosovo context indicate that not all Serbs share dehumanizing views toward Kosovo Albanians, with many potentially open to reconciliation (IRI, 2017). Future interventions could therefore explore how exposure to peace supporting outgroup members might help reduce competitive victimhood (Bar-Tal & Halperin, 2011).

Finally, our findings have important implications for post-conflict societies and peacebuilding initiatives. Meta-humanization interventions can promote reconciliation by enhancing perceptions of shared humanity, even in contexts where war-related memories are widespread and deeply ingrained. By considering how individuals with different levels of distressing memories respond to such interventions, practitioners can better tailor their approaches to reduce competitive victimhood and improve intergroup relations. These strategies could help address psychological barriers to reconciliation, ultimately supporting long-term peace and positive intergroup engagement.

Future research can expand on our findings by addressing several limitations. While our meta-humanization strategy effectively enhanced conciliatory attitudes, our experimental vignettes were fictional and created for research purposes. This approach follows established methods in experimental intergroup research (e.g., Borinca, Falomir-Pichastor, et al., 2021; Halperin et al., 2011), and the vignettes aligned with documented variations in public attitudes (Euractiv, 2018; IRI, 2017). Recent research has shown that outgroup meta-perceptions are often more negative than the outgroup’s actual perceptions, even in conflict contexts (e.g., Guvensoy et al., 2025). Future studies should therefore enhance ecological validity by developing interventions based on real-world instances of intergroup humanization, possibly using qualitative interviews or archival sources. Using actual examples of meta-humanization from similar contexts would help improve both ecological validity and reduce the need for deception in intergroup intervention research.

Our meta-humanization intervention did not directly reduce competitive victimhood among individuals with high levels of disturbing war-related memories, suggesting that stronger war-related memories may create resistance to acknowledging outgroup victimhood. Future research should explore strategies to address competitive victimhood among highly distressed individuals. Third, since disturbing war-related memories were measured rather than manipulated, causal inferences about their moderating role are limited. Future research could explore indirect priming techniques or emotion regulation interventions to test whether shifting engagement with past conflicts influences reconciliation (Halperin et al., 2011; Tam et al., 2007). Additionally, while the restricted variance in intergroup contact and peace measures reflects the post-conflict context, future studies could explore alternative response scales or nonparametric techniques.

Although our use of both correlational and experimental methods strengthens the research—particularly with the experimental manipulation of the mediator (Pirlott & MacKinnon, 2016)—longitudinal research could examine the longevity of these effects. Further, future studies should control for potentially relevant factors such as meta-prejudice, prejudice, and national identification, as variations in in-group identification can affect historical memory salience (Sahdra & Ross, 2007). Finally, since this research focused exclusively on Kosovo Albanians’ perspectives, future research should examine Serbs’ experiences to gain a more comprehensive understanding of the conflict’s impact on reconciliation. Importantly, testing these findings in other post-conflict societies, such as Cyprus and Northern Ireland, would help establish their generalizability.

### Conclusion

Our research demonstrates that disturbing war-related memories predict higher perceptions of meta-dehumanization, which is associated with greater outgroup dehumanization and, consequently, reduced conciliatory attitudes. Meta-humanization can effectively promote reconciliation by improving contact orientations and feelings of peace toward former adversaries, among individuals with both low and high levels of disturbing war-related memories, though effects are stronger for those with lower levels. Notably, while meta-humanization directly reduced competitive victimhood only among those with low levels of disturbing memories, it had an indirect effect through decreased dehumanization even for those with high levels. These findings underscore the importance of considering individual differences in post-conflict settings when aiming to understand support for intergroup reconciliation.

## Supplemental Material

sj-docx-1-psp-10.1177_01461672251338559 – Supplemental material for Ameliorating War’s Shadows: The Role of War-related Memories and Meta-humanization on Intergroup Reconciliation Supplemental material, sj-docx-1-psp-10.1177_01461672251338559 for Ameliorating War’s Shadows: The Role of War-related Memories and Meta-humanization on Intergroup Reconciliation by Islam Borinca, Sofia Stathi and Theofilos Gkinopoulos in Personality and Social Psychology Bulletin

## Notes

1. All studies were conducted in accordance with ethical principles governing research involving human participants. Ethical approval was obtained prior to conducting this research. All measures, manipulations, and exclusions in the studies have been disclosed and are reported either in the article or in the Supplemental Material. The method of determining the final sample size is described in what follows, and data collection did not continue after data analysis.

2. All key analyses were rerun using only the original five items from Voci et al. (2017). The results remained significant across all studies, indicating that the observed effects are not solely driven by the inclusion of avoidance-related items.

3. We use the term *contact orientations* to refer to participants’ openness to future intergroup contact. While related to actual contact, this construct reflects a motivational disposition rather than behavioral history, consistent with prior research (e.g. Valsecchi et al., 2024). Confirmatory factor analyses across all three studies supported the empirical distinction between contact orientations and past contact: two-factor models consistently showed better fit than one-factor models (e.g., Study 1: CFI = 0.94 vs. 0.88; RMSEA = 0.08 vs. 0.12), indicating they are related but separate constructs.

4. The results presented in this study were further validated using Python (via the statsmodels library), where mediation analysis was conducted within a structural equation modeling (SEM) framework, including all outcomes simultaneously. The findings were consistent with the original analysis, confirming the robustness of the relationships between war-related memories, meta-dehumanization, outgroup dehumanization, and conciliatory attitudes.
