# Supplementary material for: Ameliorating War’s Shadows: The Role of War-related Memories and Meta-humanization on Intergroup Reconciliation
Source: Pers Soc Psychol Bull. 2025 May 21;52(8):2418–35. doi: 10.1177/01461672251338559 (PMC13310280; doi:10.1177/01461672251338559)
Supplement: sj-docx-1-psp-10.1177_01461672251338559 – Supplemental material for Ameliorating War’s Shadows: The Role of War-related Memories and Meta-humanization on Intergroup Reconciliation [file sj-docx-1-psp-10.1177_01461672251338559.docx]

### Alternative Models (Study 1)

To examine whether the order of the mediators influenced the observed effects, we tested an alternative serial mediation model in which outgroup dehumanization preceded meta-dehumanization (rather than the reverse order). We used PROCESS Model 6 (Hayes, 2018) with 5,000 bootstrapped samples, controlling for direct and indirect contact frequency.

For contact orientations, the serial indirect effect of war-related memories through outgroup dehumanization and meta-dehumanization was non-significant, B = -0.00, SE = 0.00, 95% CI [-0.01, 0.00]. These findings suggest that reversing the order of the mediators weakens the serial pathway, highlighting the importance of meta-dehumanization preceding outgroup dehumanization in shaping intergroup contact orientations.

To further explore the order of the mediators, we tested an alternative serial mediation model in which outgroup dehumanization preceded meta-dehumanization. For feelings of peace with the outgroup, the serial indirect effect of war-related memories through outgroup dehumanization and meta-dehumanization was non-significant, B = -0.01, SE = 0.00, 95% CI [-0.01, 0.00]. These findings suggest that reversing the order of the mediators reduces the explanatory power of the model, reinforcing that meta-dehumanization plays a more central role when it precedes outgroup dehumanization rather than the other way around.

To explore an alternative mediation structure, we tested a serial mediation model in which war-related memories preceded outgroup dehumanization, with meta-dehumanization as the predictor. For contact orientations, the serial indirect effect of meta-dehumanization through war-related memories and outgroup dehumanization was non-significant, B = -0.01, SE = 0.01, 95% CI [-0.02, 0.00]. These results suggest that meta-dehumanization is more effective as a mediator than as a direct predictor in shaping contact orientations. The weak indirect effect implies that the psychological impact of meta-dehumanization on intergroup contact may not be fully transmitted through war-related memories and outgroup dehumanization in this order.

For feelings of peace with the outgroup, the serial indirect effect of meta-dehumanization through war-related memories and outgroup dehumanization was non-significant, B = -0.01, SE = 0.01, 95% CI [-0.03, 0.00]. These findings suggest that meta-dehumanization has a weaker indirect association with peace with the outgroup when war-related memories precede outgroup dehumanization, supporting that meta-dehumanization functions more effectively as a mediator rather than as a primary predictor.

To further explore the mediation process, we tested a serial mediation model in which outgroup dehumanization preceded war-related memories, with meta-dehumanization as the predictor. For contact orientations, the serial indirect effect of meta-dehumanization through outgroup dehumanization and war-related memories was non-significant, B = -0.00, SE = 0.00, 95% CI [-0.01, 0.00]. These findings suggest that the order of the mediators influences the strength of the indirect effect, with meta-dehumanization having a weaker indirect association with contact orientations when outgroup dehumanization is placed before war-related memories.

For feelings of peace with the outgroup, the serial indirect effect of meta-dehumanization through outgroup dehumanization and war-related memories was non-significant, B = 0.00, SE = 0.00, 95% CI [-0.00, 0.01]. These findings suggest that the sequential pathway from meta-dehumanization to peace with the outgroup is weak when outgroup dehumanization precedes war-related memories, highlighting that meta-dehumanization has a stronger association when positioned later in the mediation chain.

### Simple Mediation Models

To examine whether meta-dehumanization mediates the relationship between war-related memories and contact orientations, we tested a simple mediation model. The indirect effect was significant, B = -0.04, SE = 0.02, 95% CI [-0.07, -0.01]. These findings suggest that war-related memories are negatively associated with willingness for intergroup contact in part through increased perceptions of meta-dehumanization.

For peace with the outgroup, the indirect effect of war-related memories through meta-dehumanization was significant, B = -0.05, SE = 0.02, 95% CI [-0.10, -0.01]. These findings suggest that war-related memories are negatively associated with feelings of peace toward the outgroup in part through increased perceptions of meta-dehumanization.

For outgroup dehumanization, the indirect effect of war-related memories through meta-dehumanization was significant, B = 0.04, SE = 0.02, 95% CI [0.01, 0.08]. These findings suggest that war-related memories are positively associated with perceptions of meta-dehumanization, which in turn are positively linked to tendencies to dehumanize the outgroup.

For contact orientations, the indirect effect of war-related memories through outgroup dehumanization was significant, B = -0.04, SE = 0.02, 95% CI [-0.07, -0.01]. These findings suggest that war-related memories are positively associated with tendencies to dehumanize the outgroup, which in turn are negatively linked to willingness for intergroup contact.

For feelings of peace, the indirect effect of war-related memories through outgroup dehumanization was significant, B = -0.05, SE = 0.02, 95% CI [-0.09, -0.01]. These findings suggest that war-related memories are positively associated with dehumanization of the outgroup, which in turn is negatively linked to feelings of peace.

For meta-dehumanization, the indirect effect of war-related memories through outgroup dehumanization was significant, B = 0.04, SE = 0.02, 95% CI [0.01, 0.08]. These findings suggest that war-related memories are positively associated with outgroup dehumanization, which in turn is positively linked to perceptions that the outgroup dehumanizes the ingroup.

For feelings of peace with the outgroup, the indirect effect of war-related memories through contact orientations was significant, B = -0.10, SE = 0.03, 95% CI [-0.17, -0.04]. These results suggest that war-related memories are negatively associated with willingness for intergroup contact, which in turn is negatively linked to feelings of peace with the outgroup.

For meta-dehumanization, the indirect effect of war-related memories through contact orientations was significant, B = 0.04, SE = 0.02, 95% CI [0.01, 0.08]. These results suggest that war-related memories are negatively associated with willingness for intergroup contact, which in turn is positively linked to perceptions of being dehumanized by the outgroup.

### Alternative Models (Study 2)

To examine whether alternative mediation structures influenced the observed effects, we tested several alternative models using PROCESS Model 8 (Hayes, 2018) with 5,000 bootstrapped samples, controlling for direct and indirect contact frequency.

**Alternative Model 1: Contact Orientations as a Mediator and Dehumanization as an Outcome**

We tested an alternative mediation model in which Contact Orientations served as the mediator and Dehumanization was the outcome variable. The index of moderated mediation was negative for both X1, B = -0.21, SE = 0.08, 95% CI [-0.37, -0.06], and X2, B = -0.16, SE = 0.08, 95% CI [-0.34, -0.03], indicating that the indirect effect of experimental conditions on Dehumanization via Contact Orientations was moderated by War-Related Memories. However, the effect was weaker compared to the primary model, suggesting that Dehumanization functions more effectively as a mediator than an outcome.

**Alternative Model 2: Contact Orientations as a Mediator and Feelings of Peace as an Outcome**

Next, we tested a model in which Contact Orientations mediated the effect of experimental conditions on Feelings of Peace. The index of moderated mediation was significant for X1, B = 0.68, SE = 0.22, 95% CI [0.20, 1.06], and X2, B = 0.52, SE = 0.23, 95% CI [0.09, 1.01]. This suggests that Contact Orientations play a mediating role in shaping intergroup peace, with War-Related Memories moderating this effect. However, given that direct effects of experimental conditions on Feelings of Peace were non-significant, the explanatory power of this alternative model is weaker compared to the primary model.

**Alternative Model 3: Feelings of Peace as a Mediator and Dehumanization as an Outcome**

We also explored a model in which Feelings of Peace mediated the relationship between experimental conditions and Dehumanization. The moderated mediation indices were weak: X1, B = -0.09, SE = 0.05, 95% CI [-0.19, -0.00], and X2, B = -0.08, SE = 0.06, 95% CI [-0.21, 0.01]. These findings suggest that the pathway through Feelings of Peace is a less effective explanation for Dehumanization than the primary model. The weaker indirect effects indicate that while Feelings of Peace is associated with Dehumanization, it does not function as a strong mediator in this process.

**Alternative Model 4: Feelings of Peace as a Mediator and Contact Orientations as an Outcome**

Finally, we examined a model where Feelings of Peace mediated the effect of experimental conditions on Contact Orientations. The index of moderated mediation was significant for X1, B = 0.26, SE = 0.12, 95% CI [0.01, 0.48], but weaker for X2, B = 0.24, SE = 0.14, 95% CI [-0.02, 0.52], with confidence intervals that included zero. These findings suggest that while Feelings of Peace plays a role in intergroup contact orientations, its influence is weaker compared to Dehumanization as a mediator.

Across all alternative models, the original model with Dehumanization as the mediator provided the strongest explanatory power. The alternative pathways through Contact Orientations and Feelings of Peace showed weaker indirect effects, with some models failing to reach statistical significance. These findings reinforce that Dehumanization serves as a key psychological mechanism linking War-Related Memories to intergroup attitudes and behaviors, supporting the primary moderated mediation model reported in the main analyses.

### Alternative Models (Study 3)

To further examine the mediation process, we tested alternative moderated mediation models where different variables served as mediators, including **contact orientations, feelings of peace, and competitive victimhood**, with **outgroup dehumanization, feelings of peace, competitive victimhood, and contact orientations** as outcome variables. We used PROCESS Model 8 (Hayes, 2018) with 5,000 bootstrapped samples, controlling for both direct and indirect contact frequency.

#### **Contact Orientations as the Mediator**

For outgroup dehumanization, the indirect effect of experimental manipulation via contact orientations was significant at both low levels of disturbing war-related memories, B = 0.35, SE = 0.08, 95% CI [0.19, 0.50], and high levels of disturbing war-related memories, B = 0.13, SE = 0.06, 95% CI [0.03, 0.26]. The moderated mediation index was significant, index = -0.09, SE = 0.04, 95% CI [-0.17, -0.01].

For peace-related attitudes, the indirect effect of experimental manipulation via contact orientations was significant at both low levels of disturbing war-related memories, B = -0.84, SE = 0.17, 95% CI [-1.17, -0.49], and high levels of disturbing war-related memories, B = -0.32, SE = 0.13, 95% CI [-0.58, -0.06]. The moderated mediation index was significant, index = 0.23, SE = 0.10, 95% CI [0.02, 0.42].

For competitive victimhood, the indirect effect of experimental manipulation via contact orientations was significant at both low levels of disturbing war-related memories, B = 0.28, SE = 0.10, 95% CI [0.11, 0.50], and high levels of disturbing war-related memories, B = 0.11, SE = 0.06, 95% CI [0.02, 0.24]. The moderated mediation index was significant, index = -0.08, SE = 0.04, 95% CI [-0.16, -0.01].

#### **Feelings of Peace as the Mediator**

For outgroup dehumanization, the indirect effect of experimental manipulation via feelings of peace was significant at both low levels of disturbing war-related memories, B = 0.29, SE = 0.09, 95% CI [0.12, 0.46], and high levels of disturbing war-related memories, B = 0.11, SE = 0.05, 95% CI [0.01, 0.21]. The moderated mediation index was significant, index = -0.08, SE = 0.04, 95% CI [-0.16, 0.00].

For contact orientations, the indirect effect of experimental manipulation via feelings of peace was significant at both low levels of disturbing war-related memories, B = -0.48, SE = 0.14, 95% CI [-0.75, -0.19], and high levels of disturbing war-related memories, B = -0.18, SE = 0.08, 95% CI [-0.34, -0.03]. The moderated mediation index was not significant, index = 0.13, SE = 0.07, 95% CI [-0.01, 0.27].

For competitive victimhood, the indirect effect of experimental manipulation via feelings of peace was significant at both low levels of disturbing war-related memories, B = 0.20, SE = 0.08, 95% CI [0.05, 0.38], and high levels of disturbing war-related memories, B = 0.07, SE = 0.04, 95% CI [0.01, 0.16]. The moderated mediation index was not significant, index = -0.05, SE = 0.03, 95% CI [-0.13, 0.01].

#### **Competitive Victimhood as the Mediator**

For outgroup dehumanization, the indirect effect of experimental manipulation via competitive victimhood was significant at both low levels of disturbing war-related memories, B = 0.39, SE = 0.13, 95% CI [0.17, 0.68], and high levels of disturbing war-related memories, B = -0.01, SE = 0.05, 95% CI [-0.12, 0.07]. The moderated mediation index was significant, index = -0.17, SE = 0.07, 95% CI [-0.32, -0.07].

For contact orientations, the indirect effect of experimental manipulation via competitive victimhood was significant at low levels of disturbing war-related memories, B = -0.35, SE = 0.10, 95% CI [-0.55, -0.16], and at moderate levels, B = -0.10, SE = 0.03, 95% CI [-0.17, -0.03], but was non-significant at high levels, B = 0.01, SE = 0.04, 95% CI [-0.06, 0.10]. The moderated mediation index was significant, index = 0.16, SE = 0.05, 95% CI [0.07, 0.27].

For feelings of peace, the indirect effect of experimental manipulation via competitive victimhood was significant at low levels of disturbing war-related memories, B = -0.43, SE = 0.16, 95% CI [-0.77, -0.15], and at moderate levels, B = -0.13, SE = 0.05, 95% CI [-0.24, -0.03], but was non-significant at high levels, B = 0.01, SE = 0.05, 95% CI [-0.07, 0.13]. The moderated mediation index was significant, index = 0.19, SE = 0.08, 95% CI [0.06, 0.36].

### These findings suggest that the indirect effects of experimental manipulation on dehumanization, feelings of peace, and competitive victimhood via different mediators depend on levels of disturbing war-related memories. However, the observed effects were consistently weaker than in the hypothesized model, further supporting the proposed mediation order.
